# Attitudes and Practices of Healthcare Professionals and Clinical Medical Students on Contraception: A Cross-Sectional Study in Cape Coast, Ghana

**DOI:** 10.1155/2021/6631790

**Published:** 2021-03-22

**Authors:** Evans Kofi Agbeno, Joseph Osarfo, Betty Anane-Fenin, Emmanuel Kusi Achampong, Naa Adei Neequaye, Douglas Aninng Opoku, Mohammed Aliyu, Sebastian Ken-Amoah, Anthony Amanfo Ofori, Joycelyn A. Ashong

**Affiliations:** ^1^Department of Obstetrics and Gynaecology, University of Cape Coast, Cape Coast, Ghana; ^2^Ghana Health Service, Asante Mampong Municipal Hospital, Asante Mampong, Ashanti Region, Ghana; ^3^Department of Obstetrics and Gynaecology, Cape Coast Teaching Hospital, Cape Coast, Ghana; ^4^Department of Medical Education and IT, University of Cape Coast, Ghana; ^5^Princess Marie Louise Children's Hospital, Accra, Ghana; ^6^School of Public Health, Kwame Nkrumah University of Science and Technology, Kumasi, Ghana

## Abstract

**Background:**

Healthcare providers play a major role in the implementation of family planning policies. In Ghana, there has been a conscious effort to improve the knowledge of preservice and practicing health professionals on family planning. However, there have been concerns about the appropriateness of the attitudes and practices of these health cadres and, hence, their propensity to become barriers to the uptake of contraception in the general population. This study is aimed at assessing the attitudes and practices of healthcare workers and clinical-year medical students in contraceptives use, advocacy, and service provision.

**Methods:**

A cross-sectional survey was conducted among health workers and clinical-year medical students from January 1 to June 30, 2018. Variables assessed included sexual activity status, previous and current contraceptive use, and satisfaction with contraceptive use among others. Data from 400 self-administered, structured questionnaires comprising close- and open-ended questions was entered in SPSS version 22 and analysed using same. The variables assessed were presented as means, frequencies, and percentages.

**Results:**

About 58% of the respondents were sexually active. Half of the participants (50.2%) had used a form of contraception before, with condoms and other barrier methods being the most preferred (67.7%). However, only 18% of respondents were on a form of contraceptive at the time of the survey. Four out of five (82.6%) of the users of these contraceptives were satisfied with their past use. A little over half of the participants had discussed contraception with their partners. Over four-fifths of participants thought family planning was beneficial and were willing to encourage others to use a method of family planning. Majority (63.7%) of the participants had had formal training in family planning, but only 72 (18%) were actively involved in the provision of family planning services.

**Conclusions:**

Although the attitudes of the health workers and trainees toward family planning were excellent generally, only a few were using a method of contraception at the time of the survey despite the fact that most of them were sexually active. There is a need to intensify communication on behaviour change towards contraception among health professionals and clinical-year medical students in order to strengthen their role as change agents in an effort to improve community uptake.

## 1. Background

Family planning allows individuals to attain their desired number of children and determine the spacing of pregnancies, through the use of contraceptive methods and the treatment of infertility. Contraceptive methods are classified as either modern or traditional. Modern methods include female and male sterilization, intrauterine devices (IUD), implants, injectables, pill, male and female condoms, lactational amenorrhoea method (LAM), and fertility-based awareness method (FBAM). Methods such as rhythm (abstinence during fertile periods), withdrawal (coitus interruptus), and folk methods [1],, which include the use of herbs and religious charms and rituals, are grouped as traditional. Due to the high failure rates associated with the traditional methods and the lack of hygiene and scientific proof of some of them[2, 3], the modern methods have gained more prominence in recent times. In making contraceptive choices, couples balance their sexual lives, their reproductive goals, and each partner's health and safety.

Family planning is a key intervention for the reduction of maternal, neonatal, and child morbidity and mortality including stillbirths [4]. It is estimated that maintaining a birth interval of at least 3 years could prevent about 35% of maternal mortality, 13% of child mortality, and 25% of under-five mortality [5]. Data from South Africa also show that with an increase in uptake of modern contraception by 0.68% annually, 23% of births, unintended pregnancies, and abortions could be averted, preventing over two million neonatal deaths and a third of maternal deaths by 2030 [6]. In addition, the barrier methods of modern contraception give dual protection against pregnancy and sexually transmitted infections like Syphilis, Gonorrhoea, and HIV. By saving money and resources that would have been used to treat unintended pregnancies and their complications, the strain on health systems and government could also be reduced [5]. Furthermore, contraception promotes gender equality and women empowerment, as it allows women to take control of their lives, pursue higher education, and achieve fulfilling goals in life in line with the fifth sustainable development goal [7].

Although modern contraception prevalence has increased worldwide among both married and unmarried women between 2000 and 2019 (55% to 57.1% and 15.4% to 20.1%, respectively) [8], there are still concerns about its low uptake in Africa by both men and women [9].

Ghana was the third African country to adopt a comprehensive population policy in 1969, after the declaration on population by world leaders in 1968 [10]. A year later, in May 1970, The National Family Planning Programme (NFPP) was launched [11]. The long-term aim was to reduce the population growth rate of approximately 3% in 1969 to 1.7% by the year 2000, by ensuring the provision of information and making contraceptive methods safe and available to all [11]. Fifty years down the lane, Ghana has made only minimal progress in this feat, with a current population growth rate of 2.15% and a projected achievement of target in the year 2035 (“Ghana[12] (Demographics, Maps, Graphs),” n.d. [13]). The contraceptive prevalence rate (CPR) in Ghana is also as low as 22.2%, a rate which is less than half the prevalence in high-income countries like the Netherlands and Ireland. Ghana has an unmet need for family planning of 32.9% [14]. Reasons for the low contraceptive prevalence include side effects of the methods, spousal or parental disapproval, cultural and religious beliefs, and wrong perceptions such as contraception being harmful to the womb or its use being limited to married women [6, 7].

Ghana's Family Planning Costed Implementation Plan 2016–2020 [15] made preservice training as one of the main strategies to improve family planning services in the country. The training curriculum was reviewed to include topics such as long-acting reversible contraceptives (LARC), health of young people, right elements, logistics management, and health worker attitude and behaviour change issues. Training materials, equipment, and medical supplies for colleges to strengthen FP preservice training were provided, and the course content and instructional plans developed and rolled out.

Contraception implementation is health-worker led, and if a group, tasked with the implementation of a policy, does not buy in to it, outcomes are affected [16]. Healthcare providers are strategically positioned to give adequate and accurate information to their clients to eliminate misconceptions and increase acceptance of contraception. However, there is evidence to suggest that, globally, providers and trainees also fall short in knowledge about family planning and may have their biases [17–20]. Providers demonstrate bias based on clients' age, marital status, and parity. Many withhold their services from adolescents due to concerns about the hormonal methods being potential threats to their future fertility or their tendency to be promiscuous because these young women know they would not get pregnant [20, 21]. This has been identified as a major barrier to contraception usage among young unmarried women [22–24]. Passing misconceptions and inadequate information over to clients also deter them from accepting certain methods [17, 18]. In addition, experience from practice shows that inadequate counselling about the side effects of family planning methods has often created panic among contraceptive users and compelled a good number to discontinue.

There is a gap in knowledge pertaining to health workers and trainee health workers' attitudes and practices towards contraceptive use in Ghana. Literature review showed only one published study of the knowledge, perception, and use of emergency contraception (EC) among student nurses and midwives in the Northern region of Ghana [20]. While 80% of participants in this study knew the time frame for taking EC pills, more than half indicated EC use promotes promiscuity.

To help fill this gap, an assessment of attitudes and practices with respect to contraception use was conducted among health care workers and clinical-year medical students in the Central Region of Ghana. The knowledge derived is expected to help formulate behaviour change communication strategies aimed at ultimately improving the contraceptive prevalence rate. It will also serve indirectly as an evaluation of the effectiveness of preservice family planning training among medical students as the Ghana FP Costed Implementation Plan [16] draws to a close.

## 2. Methodology

### 2.1. Study Design

This was a descriptive cross-sectional study aimed at assessing the attitudes and practices of healthcare professionals and trainees to contraceptive use.

### 2.2. Study Site and Population

The study was conducted from January 1 to June 30, 2018, at the Cape Coast Teaching Hospital (CCTH) in the Central Region of Ghana. CCTH was established in 1998, and it is the largest public health facility and the only tertiary medical facility in the capital of the Central Region of Ghana. It is also the main clinical teaching site for the School of Medical Sciences of the University of Cape Coast and offers a wide range of services including family planning. The target population of this study was the health workers of the hospital (1325 in total) and the clinical-year medical students who totalled 182 during the 2017/2018 academic year. The School of Medical Sciences is a government university. Students either enter fresh from high/secondary school to do a 6 straight years or enter with a first degree preferably in the health sciences to do 4 and a half years. They are then awarded the MBChB degree after having met all relevant academic requirements. They must pass a very competitive interview to be admitted.

### 2.3. Sample Size Calculation

A sample size was estimated for the clients using a 0.05 level of significance or 95% confidence interval on a one-tail test and calculated using the Henderson and Sundaresan formula [25]:

(1) $$\begin{array}{c} n=\frac{z^{2}pq}{d^{2}}, \end{array}$$

where *n* is the sample size, *z* is the reliability coefficient at 95% confident interval (1.96), *d* is the allowable error margin of 0.05, and *p* is the proportion of health workers using contraceptives.

There was no local data on contraceptive use among health workers at the time of conduct of the study, and *p* was assumed to be 50%. Although much higher than the national CPR in 2017, 50% was used on the basis that health workers have greater knowledge of the benefits of family planning and are likely to take better to contraceptive use.

(3) $$\begin{array}{c} q=1-p\left(1–0.5=0.5\right),\\ n=\frac{\left(1.96\right)2\;\left(0.5\right)\left(0.5\right)}{\left(0.05\right)2}=\approx 384. \end{array}$$

The estimated sample size was adjusted upwards by 25% on account of an anticipated high nonresponse rate based on the fact that the study questionnaires were to be self-administered and returned. This gave a total of 480.

### 2.4. Data Collection and Sampling Procedure

Purposive sampling was used for the recruitment of the participants. Participants were eligible if they were clinical-year medical students or health workers at CCTH and gave consent. The exclusion criteria were limited to refusal to grant consent. All 182 clinical-year medical students in the 2017/2018 academic year and a total of 298 health workers were approached, and the purpose of the study was explained to them. A conscious attempt was made to approach the different health cadres. An open- and close-ended, structured questionnaire written in English was self-administered to gather demographic data and information on sexual activity status, contraceptives use, satisfaction following contraceptive use, and whether or not they had had a formal training in family planning among many others. Formal training in family planning was defined as any platform for a planned and systematic transfer of knowledge and/or skills in family planning with the objective of equipping the trainee to provide quality services competently. The questionnaire was pretested among 50 health workers in a different health facility, and appropriate changes were made to its structure before use.

### 2.5. Data Processing and Analysis

The data was coded, entered in SPSS version 22, and analysed using same. The variables assessed were presented as means, frequencies, and percentages and displayed in tables. Differences in variables assessing attitudes and practices between health workers and students were compared using chi-square analysis and Fisher's exact test. *p* value < 0.05 was deemed significant.

## 3. Results

### 3.1. Demographic Characteristics of Study Participants

Two-hundred and sixty (260) questionnaires from the health workers and 140 from medical students were retrieved, giving a response rate of 83.3% (400/480).  Table 1 shows the demographic characteristics of the study participants. Females (57.5%, 230/400) and singles (77.8%, 311/400) were in the majority. The majority of the healthcare givers (81.2%, 211/260) had practiced for less than 5 years with general nurses constituting the bulk (43.5%, 113/260) (not shown in Table 1). Participants' ages ranged from 19 to 53 years with a mean (SD) of 27.4 (5.35).

### 3.2. Participants' Attitudes towards Family Planning

The majority (63.7%, 255/400) of the participants had formal training in family planning and were sexually active (57.8%, 231/400) (see Table 2). Only a handful (18.0%, 72/400) were actively involved in the provision of family planning methods. Over four-fifths of participants found family planning beneficial, thought they will use family planning methods in the near future and will encourage others to use a method. Only about half (48.8%, 195/400) of the participants indicated ever discussing contraceptive use with their partners.

### 3.3. Contraceptive Practices among Study Participants

The contraceptive practices among the study participants are presented in Table 3.

A little over half (50.2%, 201/400) of the participants had ever used a method of contraception, with condoms and other barrier methods being the most used (67.7%, 136/201). About 16% (32/201) of those who had ever used a method of contraception indicated they were not satisfied with their particular experience.

Less than a fifth (18.0%, 72/400) of the participants were using a contraceptive method at the time of conduct of the study (current use) with half of them using condom and other barrier methods because of comfort and ease of use. Consistent use of contraceptives stood at 51.4% (37), with the intention of preventing unintended pregnancy being the main reason.

Only 12.5% (9/72) of the respondents indicated their partners were not aware they were using a contraceptive method. The reasons given were that either they did not want to have children with their partners or they felt it was within their rights and thus did not need to discuss with their partners. More than half (58.8%, 190/323) of the participants who were not using a contraceptive method at the time of the study were not willing to disclose their reasons for nonuse while 10.5% (34/323) indicated fear of side effects kept them from using contraceptives.

### 3.4. Comparison between Health Workers and Trainees in the Attitudes and Practices of Contraception

Table 4 presents the differences in attitudes and practices regarding contraceptive use between health workers and trainees. A greater proportion of the health workers were sexually active (72% vs. 31.4%; *p* < 0.001) and had used a form of contraceptive before (62.4% vs. 27.1%; *p* < 0.001) compared to the medical students.

Similar proportions of both groups had had formal training in family planning (62% vs. 65.7%; *p* = 0.459), but more workers were expectedly actively involved in the provision of family planning methods than students (72.2% vs. 19.3%; *p* < 0.001). Again, similar proportions across both groups thought family planning were beneficial (87.8% vs. 89.3%; *p* = 0.924), had discussed contraception with their partners before (52.2% vs. 42.9%; *p* = 0.203), and were satisfied with contraceptive use in the past (83% vs. 84.2%; *p* = 0.951).

While similar proportions of both groups were using a method of contraception at the time of the study (19.2% vs. 15.7%; *p* = 0.447), there were differences in the reasons adduced for the choice of that method. Availability, comfort, and ease of use of the method underline these differences. Consistent use of contraception was low in health workers and high in trainees (46.9% vs. 59.1%; *p* = 0.198), but this difference was not statistically significant.

## 4. Discussion

Improvement in contraceptive prevalence rate is key for population management and subsequent national development. Health workers are essential to this cause, but there is little or no data on their attitude to contraception in Ghana. To the best of our knowledge, our study is the first in Ghana to report attitudes and practices regarding contraception in health workers. Medical students were included as they will, in the near future, lead the charge to increase contraception acceptance. The study findings suggest a generally favourable attitude towards contraceptive use that appears to be drawn from practice and knowledge.

Majority of participants indicated FP was beneficial and that they were going to encourage others to use it. While this is very encouraging, there is still anxiety over about 12% of participants who reckoned FP was not beneficial. These health care providers, in particular, can easily dissuade people who consult them for advice on FP. The study did not explore the reasons for their stance on why FP is not beneficial, but it is reasonable to assume personal and judgmental reasons may play key roles. Over half of the health workers in a Nigerian study perceived providing contraceptives to unmarried adolescents amounted to promoting promiscuity and 44.2% reckoned married and unmarried adolescents ought to be denied contraceptives [22]. It may also be a question of deficiency in knowledge of contraceptives on the part of the health workers such that they feel uncomfortable recommending FP for others. In a survey of health care providers' knowledge and attitudes towards emergency contraception in Nigeria, over 30% of respondents incorrectly thought emergency contraceptions were abortifacients [18].

Although 58% of the respondents were sexually active, only 18% were current users of contraceptives. This is of great concern, and the reasons given for not being on contraceptives include lack of knowledge, partners' opposition, cost, and fear of side effects. It is imperative to address these issues especially because these are people in the health sector who are expected to be advocates of family planning. The fear of side effects needs further probing. It is unclear if the people who gave this reason were speaking out of previous experience or hearsay. If it is the latter, it will be considered more problematic as health workers, being agents of knowledge, are not expected to form health decisions on hearsay.

Underlying this fear could be a lack of appreciable understanding of side effects among health workers. In an Iranian study, about a third of health providers erroneously believed dry mouth caused by oral contraceptive pills was not an indication for drug discontinuation and had gone on to feed this information to users [26]. About half of the respondents in the present study had used a form of contraceptive in the past, with barrier methods dominating, and close to a fifth of them were not satisfied with their use. It is possible that this experience may be accounting for the current fear with contraceptive use, and further is research needed to resolve this finding.

About half of the participants in a sexual relationship had discussed contraceptives with their partners previously. Although encouraging, one would have expected a higher figure for a group of people expected to know better the benefits of contraceptive use. More importantly, it would be useful to know the scope of these discussions and how they are approached. The Ghana National Policy on contraception does not require partners to consent before a commodity is provided, but as much as practicable, clients are encouraged to engage their partners. Participants using any method of contraception at the time of the study had 83.3% of their partners being aware of their usage. This is likely because barrier methods were the number one choice of these users and would require partners' cooperation.

There was no difference in the proportion of health workers and students that were using a method of contraception at the time of the study, and condoms and other barrier methods were more popular although they are comparatively less effective than other forms of contraception when not used correctly and consistently [27]. The prevalent use of barrier methods observed in our study contrasts with reports from a systematic review involving data from 20 African countries including Ghana [7] that injectables, pills, and implants as more commonly preferred across Africa. While the review by Apanga and Adam [7] focused on married women and women in relationships, the vast majority of participants in the present study were single and included those still in school. Presumably, this population may have sex only occasionally compared to married folks and thus prefer barrier methods to long-term contraceptives.

The present research showed that more than half of the health workers had worked for less than 5 years and may have benefitted from family planning training in school and/or out of school. It is therefore revealing to note that more than a third of the health care workers surveyed had not had formal training in family planning methods. One may argue that this is hardly a challenge as these health workers may have work schedules that have very little to do with reproductive health (as shown by only 18% actively providing family planning services). However, health care workers are often a source of information on FP in the community and their lack of appropriate knowledge and negative attitudes may have adverse implications for women's uptake and continuous use of FP methods in the face of side effects that may occur. It is worth noting, also, that almost all participants are in their reproductive age and contraceptive use must be paramount to them if they are to enjoy safe and satisfying sex and plan their family without fear of unintended pregnancy.

About a third of the medical students had not had formal training in family planning, and this was considered an anomaly that needed investigation. It was realized that a whole class of 63 students (level 400) was yet to be trained and this could have accounted for the observation. It is worthy to note that medical students are involved in family planning service provision, albeit on a lesser scale than health workers. During their rotations in Obstetrics and Gynaecology and Community Medicine, they get hands-on practice at the family planning units at CCTH and other service centres within the Cape Coast metropolis. It is not expected that these students will all become reproductive health specialists in the future, but they can be good advocates for family planning/contraception use irrespective of whatever specialization path they take.

The study is limited by the use of a questionnaire to elicit responses. A qualitative approach would have helped to provide insights into the nuances behind some of the selected responses. The study also assumes that the responses provided by the respondents are a true reflection of reality as there are no means to verify them. Although no identifiers were used, it is possible that some respondents may not have given a true representation of their sexual activity status. Additionally, a random selection process for health workers may have led to different responses, but we are confident in the study findings because of the large sample size. Lastly, the study did not ascertain whether the participating students entered the medical school as high school or university graduates as the latter are likely to have had greater prior knowledge of family planning and contraceptives.

## 5. Conclusion

In conclusion, the study suggests that the majority of the health workers and students have a favourable attitude towards contraception use and are thus likely to be good advocates for family planning. Larger studies over a wider geographical area are needed to obtain results more representative of a national picture. Again, further research is needed to explore why health workers still allow fear of contraceptive side effects to be a barrier to use and the scope/approach of discussions people have with their partners regarding contraceptive use.

## Figures and Tables

**Table 1:** Demographic characteristics of study participants.

Variable	Frequency (*n* = 400)	Percentage (%)
*Gender*		
Male	170	42.5
Female	230	57.5
*Age (years)*		
Mean age (SD)	27.4 (±5.35)	
<20	1	0.3
20–29	294	73.5
30–39	84	21.0
40–49	14	3.5
≥50	3	0.8
Not indicated	4	1.0
*Marital status*		
Married	83	20.8
Single	311	77.8
Cohabiting	1	0.3
Divorced/separated	1	0.3
Widowed	2	0.5
Not indicated	2	0.5
*Religion*		
Christian	384	96.0
Moslem	12	3.0
Other^a^	4	1.0
*Occupation/participant's status*		
Students/trainees	140	35.0
Health workers	255	63.7
Not indicated	5	1.3
*Years of practice^b^* (*n* = 260)		
<5	211	81.2
5–9	30	11.5
10–14	11	4.2
15–19	3	1.2
≥20	5	1.9

^a^Other refers to Hindu and nonreligious people. ^b^This variable is restricted to health workers, and the denominator is the total number of health workers, i.e., 260. Not indicated = missing value.

**Table 2:** Healthcare givers' attitudes towards family planning.

Variable	Frequency (*n* = 400)	Percentage (%)
Formal training in family planning?		
Yes	255	63.7
No	145	36.3
Actively involved with the provision of family planning methods?		
Yes	72	18.0
No	328	82.0
Sexually active?		
Yes	231	57.8
No	166	41.5
Not indicated	3	0.8
Is the use of family planning beneficial?		
Yes	353	88.3
No	9	2.3
Do not know yet	38	9.5
Will you use a method of family planning?		
Yes	327	81.8
No	16	4.0
Do not know yet	57	14.2
Will you encourage someone closely related to you to use a method?		
Yes	352	88.0
No	13	3.3
Do not know yet	35	8.8
Will you encourage someone not closely related to use a method?		
Yes	358	89.5
No	10	2.5
Do not know yet	32	8.0
Have you discussed contraceptive use with your partner before?		
Yes	195	48.8
No	188	47.0
Not indicated	17	4.3

**Table 3:** Contraceptive practices among study participants.

	Frequency	Percentage (%)
*Have you used a form of contraceptive before?* (*n* = 400)		
Yes	201	50.2
No	191	47.8
Not indicated	8	2.0
*If yes, which one(s)?* (*n* = 201)		
Oral	25	12.4
Condom and other barrier methods	136	67.7
Vaginal ring	1	0.5
Injectables	14	7.0
Subdermal	9	4.5
Dermal patch	3	1.5
IUD	9	4.5
Permanent (BTL/vasectomy)	1	0.5
Natural	3	1.5
*Were you satisfied with the contraceptive used in the past?* (*n* = 201)		
Yes	166	82.6
No	32	15.9
Not indicated	3	1.5
*Are you currently on a method of contraception?* (*n* = 400)		
Yes	72	18.0
No	323	80.8
Not indicated	5	1.3
*If yes, which one?* (*n* = 72)		
Oral	8	11.1
Condom and other barrier methods	36	50.0
Injectables	8	11.1
Subdermal	4	5.6
Dermal patch	2	2.8
IUD	9	12.5
Permanent (BTL/vasectomy)	1	1.4
Natural	4	5.6
*If not using a method now, what is the reason(s)?* (*n* = 323)		
Currently pregnant	7	2.2
Desire to have a child	21	6.5
Currently in the puerperium period	3	0.9
Lack of knowledge regarding contraceptive	7	2.2
Against religious beliefs	15	4.6
Opposition of partner	7	2.2
Fear of side effects	34	10.5
Partner has a permanent method	3	0.9
Not willing to disclose	190	58.8
Too expensive	4	1.2
Single	6	1.9
Not sexually active	18	5.6
Menopause	1	0.3
Menstrual cycle	1	0.3
Other	4	1.2
Not indicated	2	0.6
*Why did you choose the method you are currently on?* (*n* = 72)		
Easily available	27	37.5
Comfortable and easy use	31	43.1
Inexpensive	6	8.3
Partner's choice	4	5.6
Other	1	1.4
Not indicated	3	4.2
*How often do use a method of contraceptive?* (*n* = 72)		
Always	37	51.4
Sometimes	32	44.4
Not indicated	3	4.2
*What is your reason for using a contraceptive?* (*n* = 72)		
Having a child when required	9	12.5
Spacing of birth	6	8.3
Prevention of unwanted births	42	58.3
Prevention of STDs	9	12.5
Improvement of health	3	4.2
Menstrual (to correct dysfunctional bleeding)	1	1.4
Not indicated	2	2.8
*Is your partner aware you are on a contraceptive method?* (*n* = 72)		
Yes	60	83.3
No	9	12.5
Not indicated	3	4.2
*If he/she is not aware, why have you not told him?* (*n* = 9)		
I do not want to have children with him	2	22.2
It is my right	3	33.3
Not willing to disclose the reason	4	44.5

**Table 4:** Comparison of attitudes and practices regarding contraceptive use between health workers and students/trainees.

Variable	Health workers *n* = 255 (%)	Trainees *n* = 140 (%)	*p* value
*Formal training in family planning?*			0.459
Yes	158 (62.0)	92 (65.7)	
No	97 (38.0)	48 (34.3)	
*Actively involved with the provision of family planning methods?*			0.001
Yes	58 (22.7)	13 (9.3)	
No	197 (77.3)	127 (90.7)	
*Sexually active?*			<0.001
Yes	184 (72.2)	44 (31.4)	
No	68 (26.7)	96 (68.6)	
Not indicated	3 (1.2)	—	
*Is the use of family planning beneficial?*			^a^0.924
Yes	224 (87.8)	125 (89.3)	
No	6 (2.4)	3 (2.1)	
Do not know yet	25 (9.8)	12 (8.6)	
*Will you use a method of family planning?*			0.092
Yes	216 (84.7)	107 (76.4)	
No	10 (3.9)	6 (4.3)	
Do not know yet	29 (11.4)	27 (19.3)	
*Will you encourage someone closely related to you to use a method?*			^a^0.454
Yes	225 (88.2)	122 (87.1)	
No	10 (3.9)	3 (2.1)	
Do not know yet	20 (7.8)	15 (10.7)	
*Will you encourage someone not closely related to use a method?*			0.491
Yes	231 (90.6)	122 (87.1)	
No	5 (2.0)	5 (3.6)	
Do not know yet	19 (7.5)	13 (9.3)	
*Have you discussed contraceptive use with your partner before?*			0.203
Yes	133 (52.2)	60 (42.9)	
No	116 (45.5)	69 (49.3)	
Not indicated	6 (2.4)	11 (7.9)	
*Have you used a form of contraceptive before?*			<0.001
Yes	159 (62.4)	38 (27.1)	
No	90 (35.3)	100 (71.4)	
Not indicated	6 (2.4)	2 (1.4)	
*If yes, which one(s)?*			0.834
Oral	20 (12.6)	3 (7.9)	
Condom and other barrier methods	105 (66.0)	29 (76.3)	
Vaginal ring	1 (0.6)	—	
Injectables	13 (8.2)	1 (2.6)	
Subdermal	7 (4.4)	2 (5.26)	
Dermal patch	3 (1.9)	—	
IUD	7 (4.4)	2 (5.26)	
Permanent (BTL/vasectomy)	1 (0.6)	—	
Natural	2 (1.3)	1 (2.6)	
*Were you satisfied with the contraceptive used in the past?*			0.951
Yes	132 (83.0)	32 (84.2)	
No	24 (15.1)	6 (15.8)	
Not indicated	3 (1.9)	—	
*Are you currently on a method of contraception?*			0.447
Yes	49 (19.2)	22 (15.7)	
No	205 (80.4)	114 (81.4)	
Not indicated	1 (0.4)	4 (2.9)	
*If yes, which one?*			^a^0.412
Oral	3 (6.1)	5 (22.7)	
Condom and other barrier methods	25 (51.0)	10 (45.5)	
Injectables	6 (12.2)	—	
Subdermal	4 (8.2)	2 (9.1)	
Dermal patch	2 (4.1)	4 (18.2)	
IUD	5 (10.2)	—	
Permanent (BTL/vasectomy)	1 (0.4)	—	
Natural	3 (2.0)	1 (4.6)	
*If not using a method now, what is the reason(s)?*			<0.001^a^
Currently pregnant	7 (3.4)	—	
Desire to have a child	19 (9.3)	1 (0.9)	
Currently in the puerperium period	3 (1.5)	—	
Lack of knowledge regarding contraceptive	5 (2.4)	2 (1.8)	
Against religious beliefs	9 (4.4)	6 (5.3)	
Opposition of partner	6 (2.9)	1 (0.9)	
Fear of side effects	24 (11.7)	8 (7.0)	
Partner has a permanent method	2 (1.0)	1 (0.9)	
Not willing to disclose	116 (56.6)	74 (64.9)	
Too expensive	1 (0.5)	3 (2.6)	
Single	4 (2.0)	2 (1.8)	
Not sexually active	4 (2.0)	13 (11.4)	
Menopause	1 (0.5)	—	
Menstrual cycle	1 (0.5)	—	
Other	2 (1.0)	2 (1.8)	
Not indicated	1 (0.5)	1 (0.9)	
*Why did you choose that method?*			0.014^a^
Easily available	21 (42.9)	6 (27.3)	
Comfortable and easy use	23 (46.9)	8 (36.4)	
Inexpensive	2 (4.1)	4 (18.2)	
Partner's choice	—	3 (13.6)	
Other	1 (2.0)	—	
Not indicated	2 (4.1)	1 (4.5)	
*How often do use a method of contraceptive?*			0.198
Always	23 (46.9)	13 (59.1)	
Sometimes	25 (51.0)	7 (31.8)	
Not indicated	1 (2.1)	2 (9.1)	
*What is your reason using a contraceptive?*			0.291^a^
Having a child when required	6 (12.5)	2 (9.5)	
Spacing of birth	5 (10.4)	1 (4.8)	
Prevention of unwanted births	26 (54.2)	16 (76.2)	
Prevention of STDs	8 (16.7)	1 (4.8)	
Improvement of health	3 (6.3)	—	
Menstrual	—	1 (4.8)	
*Is your partner aware you are on a contraceptive method?*			1.000^a^
Yes	42 (16.5)	17 (12.1)	
No	6 (2.4)	3 (2.1)	
Not indicated	207 (81.2)	120 (85.7)	
*If he/she is not aware why have you not told him?*			1.000^a^
I do not want to have children with him	1 (16.7)	1 (33.3)	
It is my right	2 (33.3)	1 (33.3)	
Not willing to disclose the reason	3 (50.0)	1 (33.3)	

Not indicated = missing numbers. ^a^Analysed using Fischer's exact test.

## Data Availability

The datasets used and/or analysed during the current study are available from the corresponding author on reasonable request.
